# Supplementary material for: Coproducing a conceptual understanding of unmet palliative care needs: stakeholder workshops using modified nominal group technique
Source: BMC Palliat Care. 2025 Dec 23;25:30. doi: 10.1186/s12904-025-01971-4 (PMC12837985; doi:10.1186/s12904-025-01971-4)
Supplement: Supplementary file 2 — Supplementary Material 2 [file 12904_2025_1971_MOESM2_ESM.docx]

**Additional Table 1 Consolidated Criteria for Reporting Qualitative Studies (COREQ) guidelines as outlined by Tong et al, 2007**

| **Domain 1: Research team and reflexivity** | | **Page number** |
| --- | --- | --- |
| 1. Interviewer | AEB, KES, AF, and JMD facilitated workshop discussions, and FEMM, TJ, AF, JMD, and LKF scribed. | Page 8 and 9 |
| 2. Credentials | Post doctoral researcher (TJ, JMD); Lecturer (AEB); Senior Research Fellow (AF); Professor (KES, FEMM, LKF, and IJH). | Page 8 |
| 3. Occupation | Researchers/academics in palliative care, part time clinicians | Page 8 |
| 4. Gender | All females | Page 8 |
| 5. Experience and training | All members of the research team are postdoctoral researchers. FEM, KES, LKF, and IJH are professors with substantial research experience. All authors have academic credentials. | Page 8 |
| 6. Relationship with participants | A relationship has been established via email with all participants prior to conducting the workshops. There was a mix of those who were unknown to the research team and those who have relationships with members of the research team through research or clinical activities. | Page 8 |
| 7. Participants knowledge | The research team introduced themselves at the beginning of the workshops and participants were informed about the purpose of the study in a participant information sheet. | Page 7 and 9 |
| 8. Interviewer characteristics | The stakeholder workshops were facilitated by four members of the research team, all of whom have experience in qualitative methods and palliative care research and were known to some participants through professional networks. Reflexivity was maintained through regular team discussions. No personal or clinical relationships existed between facilitators and participants. | Page 8 and 12 |
| **Domain 2: study design** | |  |
| 9. Methodological orientation and Theory | This study used a modified nominal group technique. Documentation from note-takers were analysed using content analysis. | Page 6 and 11 |
| 10. Sampling | We used purposive sampling. Where professional experts were interested but unable to attend, they were asked to nominate a colleague to invite. | Page 8 |
| 11. Method of approach | People with lived experience were invited via an advertisement posted on PPI forums and asked to email the research team if they were interested. People with professional experience were approached via email. | Page 7 and 8 |
| 12. Sample size | 28 participants | Page 12 |
| 13. Non-participation | People with lived experience: 20 responded to the advert registering interest. Of those, 6 did not respond to subsequent emails, two did not meet the eligibility criteria and one declined.  People with professional experience: 30 were invited via email. Of those, 13 declined or did not respond; 1 declined workshop 2 due to availability but participated in workshop 3. | Page 12 |
| 14. Setting of data collection | The workshops were conducted online via Microsoft Teams. | Page 8 |
| 15. Presence of non- participants | We had administrative support from one member of the research team who was present to support participants in joining the Microsoft Teams call and be attentive to the well-being of participants. | Page 8 and 9 |
| 16. Description of sample | Participants were described in terms of their self-reported age, gender, ethnicity, country of residence, experience and years of professional experience (where relevant). | Page 8 |
| 17. Interview guide | The workshops followed the steps detailed in Table 1 in the manuscript. | Page 10 |
| 18. Repeat interviews | The workshops followed the steps detailed in Table 1 in the manuscript. | Page 10 |
| 19. Audio/visual recording | Recordings of the workshops were used for noted taking and checking for accuracy only. | Page 9 |
| 20. Field notes | Scribes were assigned to each breakout group to document the discussions. | Page 9 |
| 21. Duration | Each workshop was 3 hours in duration. | Page 8 |
| 22. Data saturation | Participants were allowed time to discuss their points in detail and were encouraged to email with any further thoughts after the workshop if there was insufficient time to include them in the workshop. | Page 9 |
| 23. Transcripts returned | Notes from scribes were collated after each workshop for analysis. In the third workshop, the research team presented the initial list of elements of unmet palliative care needs to participants for discussion, elaboration and refinement. | Page 9 |
| **Domain 3: analysis and findings** | |  |
| 24. Number of data coders | Three people were involved in coding the data (AEB, TJ and FEMM). | Page 11 |
| 25. Description of the coding tree | Not applicable | N/A |
| 26. Derivation of themes | Themes were inductively derived from data from the workshop discussions. | Page 11 |
| 27. Software | Not applicable | N/A |
| 28. Participant checking | Participants fed back on the elements of unmet palliative care need as per the modified nominal group technique detailed in Table 1 in the manuscript. | Page 10 |
| 29. Quotations presented | Quotations are not presented. | N/A |
| 30. Data findings consistent | Yes, the findings were consistent with the data. | Page 14 |
| 31. Clarity of major themes | Major themes are presented in the results section and repeated in the conclusion. | Pages 12-19 |
| 32. Clarity of minor themes | Minor themes are also presented narratively in the results section. | Pages 13-17 |
